# Treatment Adherence and Treatment Effectiveness in Stable Chronic Obstructive Pulmonary Disease Among Outpatients at An Giang Province, Vietnam

**DOI:** 10.1155/pm/7201063

**Published:** 2026-09-06

**Authors:** Khanh Duy Dang, Lich Thanh Vu, Si Van Tran

**Affiliations:** ^1^ Department of Pharmacology and Clinical Pharmacy, Faculty of Pharmacy, Can Tho University of Medicine and Pharmacy, Can Tho City, Vietnam, ctu.edu.vn; ^2^ Department of Pharmacy, Kien Giang General Hospital, Rach Gia, An Giang Province, Vietnam; ^3^ Kien Giang General Hospital, Rach Gia, An Giang Province, Vietnam

**Keywords:** COPD assessment test (CAT), Modified Medical Research Council (mMRC) dyspnea scale, Morisky Medication Adherence Scale (MMAS-8), stable chronic obstructive pulmonary disease, treatment adherence

## Abstract

**Background:**

Recently, early prevention, treatment, and stabilization of chronic obstructive pulmonary disease (COPD) have become key priorities to reduce exacerbations and improve respiratory function. This study is aimed at evaluating treatment adherence and effectiveness among outpatients with stable COPD in Vietnam.

**Methods:**

A cross‐sectional descriptive study was conducted on 400 stable COPD outpatients at Kien Giang General Hospital (June 2024–June 2025). Treatment adherence was assessed using the 8‐item Morisky Medication Adherence Scale (MMAS‐8). Treatment effectiveness was evaluated using three criteria: the number of exacerbations and hospitalizations in the previous 12 months, the modified Medical Research Council (mMRC) dyspnea scale score, and the COPD Assessment Test (CAT) score at two time points: *T*_0_ (baseline) and T (after 3 months of outpatient follow‐up).

**Results:**

After 3 months of outpatient follow‐up, the treatment adherence rate was 76.5%. The chosen treatment regimen was significantly associated with medication adherence (*p* < 0.001). Patients using regimens (ICS/LABA + LABA [p.o.]), (SABA + ICS/LABA), and (SABA + ICS/LABA + LABA [p.o.]) had higher adherence than those using ICS/LABA alone. The overall treatment effectiveness improvement rate was 70.3%, with improvements in exacerbations/hospitalizations in the previous 12 months (23.0%), mMRC score (32.3%), and CAT score (66.0%). Although the treatment efficacy index showed positive trends for exacerbations/hospitalizations (11.0%, *p* = 0.076), mMRC score (3.1%, *p* = 0.375), and CAT score (3.5%, *p* = 0.088), these changes did not reach statistical significance, indicating only early positive trends over the 3 months. Multivariate logistic regression analysis identified treatment adherence and smoking status as the strongest predictors of treatment effectiveness.

**Conclusion:**

Patients with good adherence had substantially higher treatment effectiveness than those with poor adherence. Conversely, smoking status was negatively associated with treatment effectiveness. Further interventional studies targeting these factors are needed to improve medication adherence and treatment effectiveness in patients with stable COPD.

## 1. Introduction

Chronic obstructive pulmonary disease (COPD) is a leading cause of morbidity and mortality worldwide, including in Vietnam, and imposes an increasing socioeconomic burden. COPD ranks as the third leading cause of death globally, with 3.2 million deaths annually, accounting for 81.7% of all deaths from chronic respiratory diseases [1]. Of these, 90% occur in low‐ and middle‐income countries [2, 3]. In recent years, the focus has shifted from mere prevention and treatment of COPD to early prevention, early treatment, and disease stabilization, with interventions aimed at reducing clinical symptoms, decreasing the frequency and severity of exacerbations, and improving lung physiological function. Pharmacological treatment forms the cornerstone of COPD management, playing a critical role in alleviating symptoms, reducing exacerbation frequency and severity, and improving overall health status [4–7]. Patient adherence to prescribed medications is a decisive factor in achieving these therapeutic goals. However, COPD is a progressive chronic condition requiring long‐term, ongoing, and sometimes complex treatment regimens, leading to generally lower adherence rates compared to acute illnesses [8, 9].

Evaluating treatment effectiveness in stable COPD requires multiple clinical and paraclinical criteria to comprehensively reflect outcomes, including symptoms, respiratory function, and risk of disease progression. Achievement of at least one of the following criteria is considered an essential indicator of treatment effectiveness or improvement: Reduction in the number of exacerbations or hospitalizations in the previous 12 months compared with baseline, reflecting improved disease control and respiratory stability [10]; Reduction of at least 1 point on the modified Medical Research Council (mMRC) dyspnea scale or achievement of mild dyspnea (mMRC 0–1), indicating significant improvement in dyspnea and exercise tolerance [11]; Reduction of at least 2 points on the COPD Assessment Test (CAT) or achievement of mild symptoms (CAT < 10), representing the internationally recognized minimum clinically significant difference (MCID) for meaningful improvement in COPD‐related quality of life [12]. In clinical practice, regular monitoring of these criteria enables physicians to objectively assess treatment effectiveness and promptly adjust regimens (personalized medicine) to optimize symptom control, enhance quality of life, and maintain disease stability.

Among patients with stable COPD, limited medication adherence leads to poor symptom control, more frequent exacerbations, higher hospitalization rates, and reduced quality of life. In the Mekong Delta region, Vietnam, where smoking prevalence is high, most of the population lives in rural areas, and access to healthcare remains challenging, this issue is particularly pronounced. Based on these considerations, we conducted this study to evaluate medication adherence and treatment effectiveness in stable COPD among outpatients at Kien Giang General Hospital, Vietnam, and identify factors associated with adherence and treatment effectiveness.

## 2. Materials and Methods

### 2.1. Study Design

The research population consisted of outpatients diagnosed with and treated for stable COPD (ICD‐10 code: J44) at Kien Giang General Hospital from June 2024 to June 2025. Inclusion criteria were patients diagnosed with stable COPD based on a history of risk factor exposure, predominant clinical symptoms, and spirometry showing postbronchodilator FEV_₁_/FVC < 70%. Patients were managed and treated on an outpatient basis for at least 3 months from the initial study enrollment, were aged 40 years or older, and provided informed consent, either the patient or a family member. Excluded subjects were patients with psychiatric disorders, uncooperative patients, patients unable to respond to questions, patients with conditions affecting the performance or interpretation of pulmonary function tests, or patients with concomitant respiratory diseases (tuberculosis, asthma).

The sample size was calculated using the formula

n=Z12−α/2 p1−pd2.

Where:

*n* = sample size

*Z*1 − *α*/2 = confidence interval with 95% reliability (*α* = 0.05), and *Z* = 1.96,

*p* = expected proportion of good adherence (0.493, based on Ta′s study) [13],

*d* = *t* *o* *l* *e* *r* *a* *n* *c* *e* (0.05).

The calculated sample size was 384. In practice, 400 eligible outpatients were enrolled after applying the inclusion and exclusion criteria. Convenience sampling of outpatients with a confirmed stable COPD diagnosis was used. Patients meeting inclusion criteria and not violating exclusion criteria were consecutively enrolled until the target of 400 cases was reached at both time points: *T*_0_ (baseline, at study enrollment) and T (3 months after *T*_0_).

### 2.2. Data Collection

Medication adherence was assessed using the 8‐item Morisky Medication Adherence Scale (MMAS‐8) [14–16]. For questions 1–4 and 6–8, a “No” response scores 1 point each. For Question 5, a “Yes” response scores 1 point. The total adherence score ranges from 0 to 8. Adherence levels were categorized as high (8 points), medium (6–7 points), and low (< 6 points). Overall adherence was dichotomized into adherent (≥ 6 points) and nonadherent (< 6 points).

Factors potentially associated with medication adherence included: age, sex, residence (urban or rural), education level, smoking status, COPD duration, body mass index (BMI), number of comorbidities, number of exacerbations or hospitalizations in the previous 12 months, number of prescribed medications, treatment regimen, and inhaler device type.

Treatment effectiveness was evaluated by comparing changes from *T*_0_ to T in the number of exacerbations/hospitalizations in the previous 12 months, mMRC dyspnea score, and CAT score. Improvement in treatment effectiveness was defined as achieving at least one of the following: (1) a reduction of at least 1 exacerbation/hospitalization [10]; (2) a reduction of at least 1 point on the mMRC scale or achieving mild dyspnea (mMRC 0–1) [12]; (3) a reduction of at least 2 points on the CAT score or achieving mild symptoms (CAT < 10) [13]. This approach was selected because a single metric might overlook patients who experience symptom relief without a corresponding change in exacerbation history, or vice versa. By combining acute risk indicators with patient‐reported functional and quality‐of‐life scores, this composite measure provides a broader framework for tracking short‐term clinical responses.

The treatment efficacy index (TEI) for each criterion after 3 months was calculated as

TEI %=PM0–PMt/PM0×100.

Where:

*P* *M*_0_ = proportion of patients with at least one improvement indicator.

*P* *M*_*t*_ = proportions at *T*.

Factors potentially associated with treatment effectiveness included medication adherence, age, sex, residence, education level, smoking status, COPD duration, BMI, number of comorbidities, number of medications, and treatment regimen.

### 2.3. Ethics Statement

The study was approved by the Institutional Review Board for Biomedical Research of Can Tho University of Medicine and Pharmacy (approval number: 24.005.HV/PCT‐HĐĐĐ, dated June 28, 2024), and by the Board of Directors and the Scientific and Technical Council of Kien Giang General Hospital. The patients′ legal guardian/next of kin provided written informed consent to participate in this study.

### 2.4. Statistical Analysis

Data collection forms were entered into Microsoft Excel 2013, and statistical analysis was performed using SPSS Version 26. Comparisons of two or more proportions were conducted using *t*‐tests, ANOVA, chi‐square tests (*χ* ^2^), or nonparametric tests (e.g., Wilcoxon) as appropriate, with statistical significance set at *p* < 0.05. Univariate logistic regression was first performed to assess the unadjusted association between each independent variable and treatment effectiveness. Variables with *p*‐values < 0.20 in the univariate analysis, along with factors with well‐established clinical relevance for COPD prognosis, were selected for inclusion in the final multivariate logistic regression model.

## 3. Results

Our study analyzed 400 outpatients diagnosed with and treated for stable COPD. The mean age of participants was 66.7 ± 9.1 years. The majority of patients were elderly (78.2% aged 60 years or older) and male (98.0%). Rural residents accounted for 64.3%, nearly double the proportion of urban residents. The most common education levels were primary school (49.5%) and lower secondary school (32.2%). Mean BMI was 21.7 ± 3.4 kg/m^2^, with the largest group (52.2%) having a BMI between 18.5 and 22.9 kg/m^2^. Smoking history was reported by 91% of patients, of whom 31.7% continued to smoke at the time of the survey. The mean duration of COPD was 6.1 ± 6.7 years. The average number of exacerbations/hospitalizations in the previous 12 months was 0.9 ± 1.1. According to the GOLD 2024 classification, Group E was the most prevalent (56.5%), whereas Group A was the least common (0.8%). The highest proportion of patients had 3 comorbidities (61.5%), with a mean of 3.3 ± 2.5 (Table 1).

**Table 1:** General characteristics of the study population (*n* = 400).

Characteristics		*n*(%)	Characteristics		*n*(%)
Age	40–49	7 (1.8)	BMI (kg/m^2^)	< 18.5	65 (16.3)
	50–59	80 (20.0)		18.5–22.9	209 (52.2)
	60–69	158 (39.5)		23–24.9	66 (16.5)
	≥ 70	155 (38.7)		≥ 25	60 (15.0)
	$\left(\bar{x}\right)\pm \text{SD}=66.79.1\pm$ Min: 40; max: 94			$\left(\bar{x}\right)\pm \text{SD}=21.73.4\pm$	
				Min: 13.3; max: 36.2	

Sex	Male	392 (98.0)	Residence	Rural	257 (64.3)
	Female	8 (2.0)		Urban	143 (35.7)

Education level	Illiteracy	27 (6.8)	Smoking status	Never smoking	36 (9.0)
	Elementary	198 (49.5)		Former smoking	237 (59.3)
	Junior high school	129 (32.2)		Current smoking	127 (31.7)
	High school or above	46 (11.5)			

Duration of COPD (years)	≤ 5	230 (57.5)	Exacerbations/hospitalizations in previous 12 months	0	174 (43.5)
	> 5	170 (42.5)		≥ 1	226 (56.5)
	$\left(\bar{x}\right)\pm \text{SD}=6.16.7\pm$			$\left(\bar{x}\right)\pm \text{SD}=0.91.1\pm$	
	Min: 0; max: 40			Min: 0; max: 9	

COPD classification according to GOLD 2024	Group A	3 (0.8)	Comorbidities	< 3	154 (38.5)
	Group B	171 (42.7)		≥ 3	246 (61.5)
	Group E	226 (56.5)		$\left(\bar{x}\right)\pm SD=3.32.5\pm$	
				Min: 0; max: 9	

The mean adherence score was 6.8 ± 1.4. High adherence was observed in 46.3% of patients, whereas 53.7% had medium or low adherence (Table 2). The overall treatment adherence rate was 76.5%, with only 23.5% classified as nonadherent. Specifically, 38.8% of patients reported forgetting to take medications, 29.8% discontinued treatment when symptoms improved, and 23.0% forgot to carry medications when traveling. Rates of discontinuation due to medication‐related fatigue or nonadherence in the past 2 weeks were low (1.3% and 9.8%, respectively). Nearly all patients (97.0%) took their medications as prescribed before follow‐up visits. Although 99.0% did not find adherence burdensome, 15.8% reported difficulty remembering all prescribed medications (Table 3).

**Table 2:** Levels of medication adherence (*n* = 400).

Adherence level (MMAS‐8)	*n*(%)	Description
High adherence	185 (46.3)	Score = 8
Medium adherence	121 (30.2)	Score = 6–7
Low adherence	94 (23.5)	Score < 6
Overall adherent (≥ 6 points)	306 (76.5)	High + Medium
Overall nonadherent (< 6 points)	94 (23.5)	Low

**Table 3:** Characteristics of medication adherence behaviors (*n* = 400).

Item (MMAS‐8 questions)		*n*(%)
1. Do you sometimes forget to take your medication?	Yes	155 (38.8)
	No	245 (61.2)

2. Thinking over the past 2 weeks, were there any days when you did not take your medication?	Yes	39 (9.8)
	No	361 (90.2)

3. Have you ever cut back or stopped taking your medication without telling your doctor, because you felt worse when you took it?	Yes	5 (1.3)
	No	395 (98.7)

4. When you travel or leave home, do you sometimes forget to bring your medication?	Yes	92 (23.0)
	No	308 (77.0)

5. Did you take your medication the last time you were supposed to take it?	Yes	388 (97.0)
	No	12 (3.0)

6. When you feel like your symptoms are under control, do you sometimes stop taking your medication?	Yes	119 (29.8)
	No	281 (70.2)

7. Taking medication every day is a real inconvenience for some people. Do you ever feel hassled about sticking to your treatment plan?	Yes	4 (1.0)
	No	396 (99.0)

8. How often do you have difficulty remembering to take all your medications?	Yes	63 (15.8)
	No	337 (84.2)

The chosen treatment regimen was significantly associated with medication adherence (*p* < 0.001). Patients using regimens (ICS/LABA + LABA [p.o.]), (SABA + ICS/LABA), and (SABA + ICS/LABA + LABA [p.o.]) had higher adherence than those using ICS/LABA alone. No significant associations were found between adherence and age, sex, residence, education level, smoking status, COPD duration, BMI, number of comorbidities, number of exacerbations/hospitalizations in the previous 12 months, number of medications, or type of inhaler device (Table 4).

**Table 4:** Factors related to medication adherence.

Factors		*n*(%)		OR (95% CI)	*p*
		Adherence	Nonadherence		
Age	40–49	5 (71.4)	2 (28.6)	1.371 (0.255‐7.377)	0.713
	50–59	64 (80.0)	16 (20.0)	0.857 (0.441–1.666)	0.649
	60–69	117 (74.1)	41 (25.9)	1.201 (0.716–2.017)	0.487
	≥ 70	120 (77.4)	35 (22.6)	1	—

Sex	Male	301 (76.8)	91 (23.2)	1.985 (0.465–8.464)	0.398
	Female	5 (62.5)	3 (37.5)		

Residence	Rural	192 (74.7)	65 (25.3)	0.751 (0.458–1.233)	0.257
	Urban	114 (79.7)	29 (20.3)		

Education level	Illiteracy	18 (66.7)	9 (33.3)	1	—
	Elementary	157 (79.3)	41 (20.7)	0.522 (0.219–1.248)	0.144
	Junior high school	96 (74.4)	33 (25.6)	0.688 (0.282–1.678)	0.411
	High school or above	35 (76.1)	11 (23.9)	0.629 (0.220–1.793)	0.385

Smoking status	Never smoking	26 (72.2)	10 (27.8)	1	—
	Former smoking	184 (77.6)	53 (22.4)	0.749 (0.340–1.651)	0.474
	Current smoking	96 (75.6)	31 (24.4)	0.840 (0.365–1.933)	0.681

Duration of COPD (years)	≤ 5	174 (75.7)	56 (24.3)	0.894 (0.559–1.431)	0.642
	> 5	132 (77.6)	38 (22.4)		

BMI (kg/m^2^)	< 18.5	44 (67.7)	21 (32.3)	1	—
	18.5–22.9	163 (78.0)	46 (22.0)	0.591 (0.320–1.093)	0.094
	23–24.9	52 (78.8)	14 (21.2)	0.564 (0.257–1.239)	0.154
	≥ 25	47 (78.3)	13 (21.7)	0.580 (0.259–1.296)	0.184

Comorbidities	< 3	115 (74.7)	39 (25.3)	0.849 (0.530–1.360)	0.496
	≥ 3	191 (77.6)	55 (22.4)		

Exacerbations/hospitalizations in previous 12 months	0	129 (74.1)	45 (25.9)	0.794 (0.499–1.262)	0.328
	≥ 1	177 (78.3)	49 (21.7)		

Number of drugs in a prescription	< 5	101 (73.7)	35 (26.3)	0.794 (0.492–1.282)	0.344
	≥ 5	205 (77.9)	58 (22.1)		

Regimens	ICS/LABA	35 (52.2)	32 (47.8)	1	—
	ICS/LABA + LABA (u)	95 (79.8)	24 (20.2)	0.276 (0.143–0.532)	**< 0.001**
	SABA + ICS/LABA	88 (80.7)	21 (19.3)	0.261 (0.133–0.513)	**< 0.001**
	SABA/SAMA + ICS/LABA	1 (100.0)	0 (0.0)	—	1.000
	ICS/LABA/LAMA + LABA (u)	1 (100.0)	0 (0.0)	—	1.000
	SABA + ICS/LABA + LABA (u)	83 (83.0)	17 (17.0)	0.224 (0.110–0.455)	**< 0.001**
	SABA/SAMA + ICS/LABA + LABA (u)	3 (100.0)	0 (0.0)	—	1.000

Types of inhaler	MDI	273 (76.9)	82 (23.1)	1	—
	DPI	7 (58.3)	5 (41.7)	2.378 (0.735–7.692)	0.148
	MDI + SMI	5 (100.0)	0 (0.0)	—	1.00
	DPI + MDI	19 (73.1)	7 (26.9)	1.227 (0.498–3.020)	0.657
	DPI + SMI	1 (100.0)	0 (0.0)	—	1.000
	ELLIPTA	1 (100.0)	0 (0.0)	—	1.000

*Note:* *p* < 0.001 compared to the ICS/LABA group. *p* < 0.001 for the regimens ICS/LABA + LABA (u), SABA + ICS/LABA, and SABA + ICS/LABA + LABA (u) represent significant differences in adherence when compared directly to the reference regimen, ICS/LABA.

After 3 months of outpatient follow‐up, 23.0% of patients showed improvement in exacerbations/hospitalizations, whereas 77.0% did not. Higher improvement rates were observed for mMRC (32.3%) and CAT (66.0%) scores. The overall improvement rate in treatment effectiveness was 70.3% (Table 5). The proportion of patients with at least one exacerbation or hospitalization in the previous 12 months decreased from 56.5% to 50.3%, suggesting a marginal statistical trend (TEI) of 11.0% (*p* = 0.076). Similarly, the reductions in the proportions of patients with mMRC ≥ 2 (*p* = 0.375) and CAT ≥ 10 (*p* = 0.088) were not statistically significant. These changes should be interpreted strictly as observational trends rather than definitive clinical improvements (Table 6).

**Table 5:** Criteria for evaluating treatment effectiveness in stable COPD (*n* = 400).

Criterion	Number of patients improved n (%)
Reduction in exacerbations/hospitalizations in the previous 12 months	92 (23.0)
Reduction in mMRC score (≥ 1 point)	129 (32.3)
Reduction in CAT score (≥ 2 points)	264 (66.0)
Overall treatment effectiveness improvement (achieving at least one of the above criteria)	281 (70.3)

**Table 6:** Treatment efficacy index between time points *T*_0_ and T.

Criterion		*n*(%)		TEI	*p*
		*T*_0_	T		
Exacerbations/hospitalizations in previous 12 months	≥ 1	226 (56.5)	201 (50.3)	11.0%	0.076
	0	174 (43.5)	199 (49.7)		

mMRC score	≥ 2	326 (81.5)	316 (79.0)	3.1%	0.375
	0–1	74 (18.5)	84 (21.0)		

CAT score	≥ 10	375 (93.8)	362 (90.5)	3.5%	0.088
	< 10	25 (6.2)	38 (9.5)		

Medication adherence and the selected treatment regimen were significantly associated with treatment effectiveness. Other factors showed no significant association. Multivariate logistic regression confirmed that treatment adherence and smoking status were the strongest predictors of treatment effectiveness (*p* < 0.001; *p* = 0.043 and *p* = 0.012, respectively) (Table 7).

**Table 7:** Factors related to treatment effectiveness in stable COPD (*n* = 400).

Factors		Treatment effectiveness *n* (%)		Univariate		Multivariate	
		Improved	Unimproved	OR (95% CI)	*p*	OR (95% CI)	*p*
Medication adherence	Adherence	277 (90.5)	29 (9.5)	214.914 (73.563–627.867)	**< 0.001**	296.482 (87.371–1.006.072)	**< 0.001**
	Nonadherence	4 (4.3)	90 (95.7)				

Age	40–49	4 (57.1)	3 (42.9)	1.892 (0.407–8.800)	0.416	2.122 (0.183–24.666)	0.548
	50–59	60 (75.0)	20 (25.0)	0.841 (0.455–1.555)	0.581	0.559 (0.179–1.748)	0.317
	60–69	106 (67.1)	52 (32.9)	1.238 (0.764–2.003)	0.386	1.040 (0.441–2.451)	0.929
	≥ 70	111 (71.6)	44 (28.4)	1	—	1	—

Sex	Male	277 (70.7)	115 (29.3)	2.409 (0.592–9.796)	0.245	1.141 (0.096–13.625)	0.917
	Female	4 (50.0)	4 (50.0)				

Residence	Rural	178 (69.3)	79 (30.7)	0.875 (0.557–1.374)	0.562	1.035 (0.454–2.355)	0.935
	Urban	103 (72.0)	40 (28.0)				

Education level	Illiteracy	17 (63.0)	10 (37.0)	1	—	1	—
	Elementary	144 (72.7)	54 (27.3)	0.637 (0.275–1.479)	0.294	1.363 (0.239–7.764)	0.727
	Junior high school	85 (65.9)	44 (34.1)	0.880 (0.372–2.083)	0.771	1.977(0.332–11.789)	0.454
	High school or above	35 (76.1)	11 (23.9)	0.534 (0.190–1.503)	0.235	0.395 (0.045–3.448)	0.401

Smoking status	Never smoking	20 (55.6)	16 (44.4)	1	—	1	—
	Former smoking	170 (71.7)	67 (28.3)	0.493 (0.241–1.008)	0.052	0.292 (0.089–0.962)	**0.043**
	Current smoking	91 (71.7)	36 (28.3)	0.495 (0.231–1.060)	0.07	0.183 (0.048–0.691)	**0.012**

Duration of COPD (years)	≤ 5	161 (70.0)	69 (30.0)	0.972 (0.630–1.501)	0.899	1.162 (0.534–2.530)	0.704
	> 5	120 (70.6)	50 (29.4)				

BMI (kg/m^2^)	< 18.5	39 (60.0)	26 (40.0)	1	—	1	—
	18.5–22.9	150 (71.8)	59 (28.2)	0.590 (0.330–1.054)	0.075	0.693 (0.244–1.972)	0.492
	23–24.9	47 (71.2)	19 (28.8)	0.606 (0.293–1.256)	0.178	0.903 (0.254–3.220)	0.876
	≥ 25	45 (75.0)	15 (25.0)	0.500 (0.232–1.076)	0.076	0.483 (0.117–1.994)	0.315

Comorbidities	< 3	105 (68.2)	49 (31.8)	0.852 (0.550–1.320)	0.474	1.759 (0.315–9.819)	0.520
	≥ 3	176 (71.5)	70 (28.5)				

Number of drugs in a prescription	< 5	91 (66.4)	46 (33.6)	0.760 (0.487–1.187)	0.227	0.461 (0.079–2.681)	0.388
	≥ 5	190 (72.2)	73 (27.8)				

Regimens	ICS/LABA	32 (47.8)	35 (52.2)	1	—	1	—
	ICS/LABA + LABA (u)	84 (70.6)	35 (29.4)	0.381 (0.205–0.709)	**0.002**	1.011 (0.316–3.241)	0.985
	SABA + ICS/LABA	82 (75.2)	27 (24.8)	0.301 (0.158–0.575)	**< 0.001**	0.670 (0.189–2.379)	0.535
	SABA/SAMA + ICS/LABA	1 (100.0)	0 (0.0)	—	1.000	—	1.000
	ICS/LABA/LAMA + LABA (u)	1 (100.0)	0 (0.0)	—	1.000	—	1.000
	SABA + ICS/LABA + LABA (u)	78 (78.0)	22 (22.0)	0.258 (0.131–0.506)	**< 0.001**	0.521 (0.146–1.852)	0.313
	SABA/SAMA + ICS/LABA + LABA (u)	3 (100.0)	0 (0.0)	—	1.000	—	1.000

*Note:* *p* < 0.05, *p* < 0.01, or *p* < 0.001 compared to adherence group, never smoking group, or ICS/LABA group. (1) Medication adherence (univariate and multivariate analysis): univariate analysis (*p* < 0.001) and multivariate analysis (*p* < 0.001). The bolded p‐values indicate that patients with adherence have a significantly higher probability of treatment effectiveness compared to the nonadherence group. (2) Smoking status (multivariate analysis): Former smoking (*p* = 0.043): the bolded p‐value indicates a statistically significant difference in treatment effectiveness for former smokers when compared directly to the never smoking reference group (OR = 1). Current smoking (*p* = 0.012): the bolded p‐value indicates a statistically significant difference in treatment effectiveness for current smokers when compared directly to the never smoking reference group (OR = 1). (3) Regimens (univariate analysis): ICS/LABA + LABA (u) (*p* = 0.002), SABA + ICS/LABA (*p* < 0.001), and SABA + ICS/LABA + LABA (u) (*p* < 0.001): the bolded p‐values show that these specific treatment regimens have a statistically significant relationship with treatment effectiveness when compared directly against the ICS/LABA reference regimen (OR = 1).

## 4. Discussion

High adherence was observed in 46.3% of patients. These results are consistent with the findings of Ta et al. (49.3% high adherence) and Montes de Oca et al. (51.0%) [13, 17]. Additionally, the overall treatment adherence rate in our study was 76.5%. This figure aligned with Moradkhani et al. (74.0%) but is lower than that reported by Montes de Oca et al. (80.1%) [17, 18]. Nevertheless, 23.5% of patients in our study remained nonadherent, underscoring that patient education, particularly during the stable phase of COPD, remains a significant challenge. Some patients may perceive the disease as stabilized and discontinue medications on their own, or face difficulties maintaining long‐term treatment without timely support from the healthcare system [8].

Forgetting to take medications was the most common reason for poor adherence in our cohort. This pattern reflects that COPD patients in our study were predominantly elderly, frequently had multiple chronic comorbidities, and were prescribed an average of 6–7 medications per day. These factors make it particularly challenging for older patients to adhere consistently to their treatment regimens. Consequently, support from family members and regular reminders from healthcare providers are essential to improve adherence. Previous studies have also shown that forgetting to take medications is the most common nonadherent behavior, accounting for 30%–50% of cases [19]. Bourbeau et al. further emphasized that a lack of understanding of the chronic nature of COPD often leads patients to discontinue treatment once symptoms improve, a pattern observed in both newly diagnosed and long‐term patients [8].

Compared with the group using ICS/LABA alone, patients on combination regimens (ICS/LABA + LABA [p.o.]), (SABA + ICS/LABA), and (SABA + ICS/LABA + LABA [p.o.]) showed significantly higher adherence (79.8%, 80.7%, and 83.0%, respectively), with all differences statistically significant. This suggests that combination regimens, particularly those incorporating as‐needed SABA alongside maintenance controller therapies, may provide superior symptom control, reduce the frequency of dyspnea and exacerbations/hospitalizations, and enable patients to perceive treatment benefits, thereby directly sustaining motivation for adherence. These results align with recent studies; however, those studies also note that using multiple inhaler devices or combining inhaled and oral medications can improve disease control but may increase the risk of reduced adherence if the regimen becomes overly complex. Indeed, COPD patients using multiple inhaler devices have a higher risk of missing doses and using incorrect technique than those using a single device, particularly among the elderly [20]. Another study reported that when regimens combine oral medications with multiple inhaled therapies, adherence depends heavily on patients′ perception of treatment benefits and the level of support provided by healthcare professionals [21, 22]. In addition, triple therapy delivered via a single inhaler device improves both adherence and treatment persistence compared with multidevice triple therapy, owing to fewer handling steps and greater convenience [23, 24].

The overall improvement rate reached 70.3%, reflecting a positive trend in the treatment of stable COPD. This figure is comparable to that reported by Boven et al, who found that patients with adherence above 80% achieved similar improvement rates [25]. However, the low improvement rate in exacerbations indicates that controlling the risk of disease progression remains a significant challenge. This underscores the need for a dual approach: symptom reduction and quality‐of‐life enhancement must be pursued in parallel with strategies to prevent exacerbations. These strategies include pharmacological optimization, pulmonary rehabilitation, influenza and pneumococcal vaccination, and comprehensive management of risk factors [7, 26].

The proportion of patients with at least one exacerbation or hospitalization in the previous 12 months decreased from 56.5% at *T*_0_ to 50.3% at T. However, this difference was not statistically significant. This finding reflects a positive trend toward better exacerbation control, although the magnitude of improvement was insufficient to achieve high statistical confidence. The proportion of patients with mMRC ≥ 2 decreased from 81.5% at *T*_0_ to 79.0% at T, but the change was not statistically significant. The modest improvement observed in this study may be attributed to the relatively short intervention duration or to patients not yet having fully implemented complementary supportive measures in a coordinated manner. The proportion of patients with a CAT score ≥ 10 decreased from 93.8% at *T*_0_ to 90.5% at T. Although this change did not reach statistical significance, it suggests a positive trend in overall symptom burden, indicating improved quality of life. Compared with the study by Wu et al, the improvement in the CAT score appears more pronounced in settings with close management and sustained interventions [27].

Among patients with good treatment adherence, favorable treatment effectiveness was observed in more than 90%. In contrast, among the nonadherent group, 95.7% showed no improvement, whereas only 4.3% improved. These findings clearly indicated that adherence decisively influences treatment effectiveness. The strong association between treatment adherence and efficacy underscores the role of adherence as a key determinant in controlling COPD. Proper adherence to the prescribed regimen helps reduce exacerbation frequency, improve symptoms, and lower hospitalization rates. Turégano‐Yedro et al. also emphasized the critical role of adherence, particularly when patients receive proper medication instruction and participate in pulmonary rehabilitation programs [28]. These results confirmed that treatment adherence is not merely an individual behavior but is significantly influenced by supportive factors from the healthcare system and the patient′s living environment.

The regimens (SABA + ICS/LABA), (SABA + ICS/LABA + LABA [p.o.]), and (SABA/SAMA + ICS/LABA + LABA [p.o.]) achieved high treatment‐effectiveness improvement rates (≥ 75%), with statistically significant differences. These results suggested that combination regimens tend to provide superior treatment effectiveness compared with ICS/LABA alone. The regimens (SABA + ICS/LABA) and (SABA + ICS/LABA + LABA [p.o.]) were more effective at improving symptoms and reducing exacerbations, owing to the additive bronchodilator effects of SABA and LABA combined with ICS for airway inflammation control [6, 29]. Notably, the regimens incorporating SABA/SAMA (SABA/SAMA + ICS/LABA and SABA/ + ICS/LABA + LABA [p.o.]) achieved 100.0% improvement. This represents an ideal combination that delivers simultaneous rapid‐acting bronchodilation (SABA, SAMA) and long‐acting bronchodilation (LABA, LAMA), thereby enhancing airflow and reducing inflammation [30]. However, the use of multiple medications in these regimens may complicate treatment adherence, particularly when patients are required to use several different devices. This could explain why the ICS/LABA‐only group showed lower improvement rates than the more complex regimens. Lipson et al. demonstrated that triple therapy (ICS/LABA/LAMA) effectively reduces exacerbations and improves lung function [31]. Similarly, Duan et al. reported that multidrug regimens can more effectively alleviate symptoms, especially in patients with severe symptoms and frequent exacerbations during the year [6]. In our study, the predominant use of complex combination regimens reflects the clinical reality that most enrolled outpatients fell into GOLD Groups B and E (99.2% combined), necessitating escalated controller therapies. However, potential prescribing biases cannot be entirely ruled out, as regimen selection in this real‐world setting is often driven by drug availability in the hospital′s provincial formulary and insurance coverage policies rather than by strictly individualized clinical trials. This baseline prescription pattern might partially confound direct comparisons of adherence across therapeutic groups.

Multivariate logistic regression analysis revealed that treatment adherence and smoking status were the two factors most strongly associated with treatment effectiveness. Patients in the adherent group had a substantially higher likelihood of achieving treatment improvement than those in the nonadherent group. Conversely, smoking status was negatively associated with treatment effectiveness. The probability of improvement was significantly lower in both smoking groups than in never‐smokers. These findings are consistent with previous studies by Agrawal et al. and Alwafi et al., which demonstrated that good treatment adherence significantly reduces hospitalization frequency and mitigates the risk of disease progression [32, 33]. Similarly, Lipson et al. emphasized that smokers exhibited markedly poorer treatment effectiveness, underscoring the need for robust smoking cessation support strategies to enhance overall outcomes [31]. Treatment adherence and smoking status emerged as the factors with the most substantial impact on treatment effectiveness. These results highlight the critical importance of improving medication adherence and providing strong smoking cessation support in the comprehensive management of COPD.

However, the multivariate analysis yielded a strikingly large adjusted odds ratio for medication adherence (OR = 296.482, 95% CI: 87.371–1006.072). This extreme value and the accompanying wide confidence interval must be interpreted with caution, as they reflect a mathematical artifact known as quasi‐complete separation or sparse‐data bias in the logistic regression model. Severe data skewness upon cross‐tabulation supports this: among the nonadherent patients (*n* = 94), 95.7% (*n* = 90) showed no clinical improvement, leaving only four patients (4.3%) who improved despite nonadherence. This severe asymmetry and sparse cell count destabilized the maximum‐likelihood estimation, inflating the odds ratio. Although this underscores the paramount deterministic clinical role of medication adherence in achieving therapeutic success in stable COPD, the exact magnitude of the OR should be viewed as an indicator of a powerful, near‐absolute association rather than a precise point estimate.

## 5. Limitations

This result reaffirms the decisive role of treatment adherence in controlling stable COPD. It aligns with the GOLD 2024 recommendations, which emphasize personalized treatment regimens, patient education, and close monitoring to reduce symptoms, improve quality of life, and prevent exacerbations. However, the cross‐sectional descriptive design, combined with a short‐term follow‐up (only 3 months), limits causal inference and may not fully capture long‐term adherence dynamics and clinical trajectories. Furthermore, our reliance on patient‐reported outcome measures, such as the MMAS‐8, mMRC, and CAT scores, introduces potential recall and social desirability biases, as patients might overreport their adherence. Additionally, several critical confounding variables were not collected, including patients′ socioeconomic status, documented inhaler technique, and biological markers such as blood eosinophils. Socioeconomic challenges in rural areas of the Mekong Delta might directly restrict access to medication, whereas suboptimal inhaler techniques could impair clinical efficacy despite high self‐reported adherence. Moreover, the extreme polarization of treatment success between the adherent and nonadherent cohorts led to quasi‐complete separation in the multivariate logistic regression model, resulting in a highly inflated odds ratio and a wide confidence interval for the medication adherence variable, which limits the precision of this specific estimate. Future longitudinal studies with extended follow‐up and multicenter data are warranted to minimize these residual confounders.

## 6. Conclusion

To enhance COPD management in community settings, multimodal interventions are recommended, including patient and family education on proper inhaler technique, adherence reminders (via mobile applications or healthcare personnel), intensified smoking cessation support, and prioritization of convenient combination regimens to improve adherence. In low‐resource and rural settings such as the Mekong Delta, cost‐effective strategies such as training community health workers to conduct regular inhaler technique audits, providing simplified medication counseling, and using basic SMS‐based adherence reminders are highly practical. Randomized controlled interventional studies with long‐term follow‐up (≥ 6–12 months) that also evaluate factors such as inhaler technique, detailed comorbidities, and blood eosinophil levels will be essential to confirm and optimize these strategies in the Vietnamese context.

**Nomenclature**
BMI: body mass index
CAT: COPD Assessment Test
COPD: chronic obstructive pulmonary disease
DPI: dry powder inhaler
FEV_1_
: forced expiratory volume in first second
GOLD: Global Initiative for Chronic Obstructive Lung Disease
ICS: inhaled corticosteroids
LABA: long‐acting *β*_2_ agonist
LAMA: long‐acting muscarinic antagonist
MDI: metered‐dose inhaler
mMRC: modified Medical Research Council
SABA: short‐acting *β*_2_ agonist
SAMA: short‐acting muscarinic antagonist

## Author Contributions

K.D.D.: study conception and design, data collection, analysis and interpretation of results, original draft preparation, review and editing; L.T.V.: study conception and design, data collection, analysis and interpretation of results, review and editing; S.V.T.: study conception and design, data collection, analysis and interpretation of results, review and editing.

## Funding

No funding was received for this manuscript.

## Disclosure

All authors have read and approved the final version of the manuscript.

## Conflicts of Interest

The authors declare no conflicts of interest.

## Data Availability

Data will be available upon reasonable request through the corresponding author.
